# Suppression of TP Rat Pancreatic Acinar Cell Apoptosis by hucMSC-Ex Carrying hsa-miR-21-5p via PTEN/PI3K Regulation

**DOI:** 10.1155/sci/8883585

**Published:** 2025-03-17

**Authors:** Zhao Zhirong, Jiang Kexin, Yuan Mu, Zhou Lichen, Tan Zhen, Liang Hongyin, Dai Ruiwu

**Affiliations:** ^1^General Surgery Center, General Hospital of Western Theater Command, Chengdu, Sichuan Province, China; ^2^Department of General Surgery, Jinling Hospital, Affiliated Hospital of Medical School, Nanjing University, Nanjing, Jiangsu Province, China; ^3^The General Hospital of Western Theater Command, Affiliated Hospital of Southwest Jiaotong University, Chengdu 610031, Sichuan, China; ^4^Pancreatic Injury and Repair Key Laboratory of Sichuan Province, General Hospital of Western Theater Command, Chengdu, Sichuan Province, China

**Keywords:** apoptosis, exosomes, pancreatitis, PTEN, stem cells

## Abstract

**Objective:** The traumatic pancreatitis (TP) has an alarmingly high mortality rate. Our previous research has demonstrated that human umbilical cord mesenchymal stem cells-derived exosomes (hucMSC-Exs) could treat TP by inhibiting acinar cell apoptosis. Accordingly, the objective of this study is to unravel the intricate mechanism behind the repair of pancreatic injury in TP rats.

**Methods:** A gene interaction network of miRNA was constructed based on the Gene Expression Omnibus (GEO) database (GSE 159814). Our investigation was divided into two groups, and appropriate controls were implemented for each group. The expression levels of inflammatory factors in each group were detected, along with the pathological damage of pancreatic tissue, the percentage of apoptotic cells, and key mRNA and protein expression levels.

**Results:** The miRNA–mRNA gene interaction network suggests that hsa-miR-21-5p/phosphatase and tensin homolog (PTEN) are positioned at the core of this interaction network. Enzyme-linked immunosorbent assay (ELISA) and histological examination (HE) results suggest that pancreatic damage increased in the miR-21 inhibitor and EXW groups, whereas it decreased in the miR-21 activator and EXC groups compared to the EX group. PCR, western blot (WB), and TdT-mediated dUTP Nick-End Labeling (TUNEL) results indicate that hucMSC-Ex carrying hsa-miR-21-5p suppresses excessive activation of PTEN by phosphoinositide 3-kinase (PI3K), exerting therapeutic effects.

**Conclusion:** This study has discovered that hucMSC-Ex effectively inhibits the translation of PTEN via the transported hsa-miR-21-5p, consequently affecting the PI3K/serine–threonine kinase (AKT) signaling pathway. This results in reduced inflammation and inhibition of acinar cell apoptosis by regulating pancreatic enzyme leakage, thereby providing a therapeutic effect on TP.

## 1. Introduction

Traumatic pancreatitis (TP) has a relatively low incidence rate among abdominal injuries, but it is associated with a staggering mortality rate of 20%, posing a grave threat to human health [1, 2]. In recent years, researchers have developed a strong interest in the therapeutic potential of human umbilical cord mesenchymal stem cells (hUC-MSCs) due to their ease of handling, storage, and transport, as well as their significantly smaller size compared to parental cells [3–5]. As a result, hUC-MSCs have been widely utilized in the treatment of traumatic diseases. Our preliminary study demonstrated that hUC-MSCs and hUC-MSCs-derived exosomes (hucMSC-Exs) could be enriched at the site of pancreatic injury in a rat model of TP via tail vein injection [6, 7]. Further analysis revealed that treatment with hucMSC-Ex reduced pathological damage in pancreatic tissue, lowered serum levels of inflammatory mediators, and prevented apoptosis of acinar cells in rats with TP. Nonetheless, the precise active components within hucMSC-Ex that contribute to these therapeutic effects are still not fully understood, and further investigation into the downstream signaling pathways is necessary.

Research has demonstrated that hucMSC-Ex plays a pivotal role in the treatment of traumatic diseases by inhibiting inflammatory responses, alleviating oxidative stress, and regulating immune reactions [8, 9]. In this complex process, miRNAs, the most enriched component of the exosomes, play an irreplaceable role [10–13]. Therefore, investigating the specific mechanisms through which hucMSC-Ex loaded with miRNAs treat TP could deepen our understanding of stem cell-derived exosomes, miRNAs, and TP. An investigation into the effects of hucMSC-Ex on acute traumatic spinal cord injury indicated its therapeutic potential by inducing axonal growth [14]. To further investigate the content and diversity of miRNAs carried by hucMSC-Ex, researchers conducted absolute quantitative sequencing, revealing that hsa-miR-21-5p is the most abundant miRNA (GSE159814) [14]. Similarly, we performed high-throughput sequencing of miRNAs extracted from hucMSC-Ex in this research and identified that hsa-miR-21-5p was notably overexpressed.

Numerous studies have documented that hsa-miR-21-5p has a direct impact on phosphatase and tensin homolog (PTEN) translation, which in turn regulates the PTEN/phosphoinositide 3-kinase (PI3K)/serine–threonine kinase (AKT) signaling pathway [15–17]. A study on the impact of miRNA following small glial cell virus infection found that high levels of hsa-miR-21-5p can inhibit PTEN, activating PI3K/AKT signaling and regulating inflammation [15]. Activation of the PI3K pathway has been shown to inhibit cell death [18, 19]. Moreover, research on esophageal cancer revealed that hsa-miR-21-5p can stimulate the PI3K pathway by suppressing PTEN expression, intensifying AKT phosphorylation, and exerting downstream effects [20]. These studies uniformly demonstrate that PTEN is a downstream target of hsa-miR-21-5p, underscoring its potential clinical value as a therapeutic target for treating diseases.

The objective of this study is to elucidate the regulatory effect of hucMSC-derived exosomes on PTEN using agomir and antagomir reagents targeting hsa-miR-21-5p. Additionally, rat models of PTEN/PI3K overexpression and inhibition were established using wortmannin (a PI3K inhibitor) and 1,3-di-caffeoylquinic acid (a PI3K agonist), respectively, to investigate the significance of the PTEN/PI3K pathway in hucMSC exosome therapy during TP.

## 2. Materials and Methods

### 2.1. Construction of the miRNA Interaction Network Carried by hucMSC-Ex

First, absolute quantification sequencing results of miRNA derived from hucMSC-Ex were extracted from the Gene Expression Omnibus (GEO) database (GSE159814). From a pool of ~990 miRNAs, we further screened and selected the top 10 ranked miRNAs. To predict the target mRNAs of the selected miRNAs, we utilized miRDB (https://mirdb.org/)—a highly reliable online tool for miRNA target prediction. The resulting data were then imported into Cytoscape (version 3.9.1) for visual representation. Through our analysis, we identified the main miRNAs and downstream targets responsible for the therapeutic effects of hucMSC-Ex. Notably, we observed that hsa-miR-21-5p had the highest abundance and directly regulated PTEN—an important tumor suppressor gene.

### 2.2. Obtaining and Identifying hUC-MSCs and hucMSC-Ex

To accurately identify the surface antigen profile of hUC-MSCs using flow cytometry, third to fifth passage hUC-MSCs were thawed and prepared as a cell suspension. The cells were divided into four groups, each resuspended in 100 μL of LBS in an EP tube. CD45 (5 μL per group), CD29 (5 μL per group), CD44 (5 μL per group), and CD34 (5 μL per group) were added to the respective tubes. The samples were incubated at 4°C in the dark for 30 min, followed by flow cytometry analysis (Beckman Coulter, Inc., USA).

The supernatant of hUC-MSCs was thawed and centrifuged at 10,000 × *g* for 45 min. The supernatant was filtered through a 0.45 μm filter and transferred to a new tube. The filtrate underwent centrifugation at 100,000 × *g* for 70 min, followed by resuspension of the pellet and a second centrifugation, discarding the supernatant. The final pellet was resuspended in 200 μL of PBS to obtain hucMSC-Ex.

Transmission electron microscopy (Hitachi, Ltd., Japan) was used to assess the morphological characteristics of exosomes. The size analysis of extracellular vesicles was performed following calibration with standard samples: 10 μL of hucMSC-Ex was diluted to 30 μL and analyzed using a particle size analyzer (Beckman Coulter, Inc., USA). Finally, the size and concentration data of hucMSC-Ex were collected.

### 2.3. Establishment of TP Rat Models for Each Group

Reagents preparation: hsa-miR-21-5p agomir and angtagomir were prepared in a ratio of 1 mg: 200 μL PBS. Wortmannin and 1,3-dicaffeoylquinic acid were dissolved in dimethyl sulfoxide and then diluted with corn oil (wortmannin concentration of 0.3 mg/mL, the 1,3-dicaffeoylquinic acid concentration of 6 mg/mL, and injection dose of 0.5 mL per rat).

A total of 35 Sprague-Dawley (SD) male rats (weighing 200 ± 25 g) were randomly divided into seven groups (five rats per group), and the sampling method was the same as before: control group, TP group, EX group, miR-21 inhibitor group, miR-21 agonist group, EXW group, and EXC group. The TP group rats were modeled in the same way as in the previous research, with an impact pressure of 3 cm^2^/12 kg (the pancreas is located deep in the abdomen, and TP patients often have high impact pressure, so 400 kPa is appropriate).

Each of the seven groups was treated differently, as shown in Table 1.

To separately investigate the effects of hsa-miR-21-5p and PTEN/PI3K on TP, we divided the study into two groups: the Control, TP, EX, miR-21 inhibitor, and miR-21 agonist groups and the Control, TP, EX, EXW, and EXC groups.

According to previous research findings, the level of apoptosis in acinar cells of pancreatic tissue in the TP model of SD rats reaches its peak 1 day after the injury [21]. Thus, the sampling time was set for 1 day postmodeling. The specific sampling method involved anesthetizing and fixing the rats, followed by opening the abdomen and collecting blood from the abdominal aorta. Then, separate the pancreas and store it appropriately.

### 2.4. The Histological Examination (HE) Staining and Pathological Scoring of Pancreatic Tissue

Pancreatic tissue samples were fixed in formaldehyde, followed by gradient dehydration using ethanol and paraffin embedding. After embedding and sectioning, the slides were hydrated and stained with hematoxylin and eosin for 10–20 min. The slides were then rinsed, dehydrated, cleared, and sealed. The pathological morphology was observed under a light microscope. Two professional pathologists evaluated the degree of pancreatic edema, hemorrhage, cell necrosis, and inflammatory cell infiltration according to the report by Schmidt et al. [22], without knowledge of the specific grouping, by selecting the area to be observed and collecting 100 × and 400 × images. The average pathological score of 10 high-power fields was considered as the final score for each slide.

### 2.5. The Enzyme-Linked Immunosorbent Assay (ELISA) Testing of Serum Index

Rat blood samples were collected and promptly cooled on ice. Within 2 h, the samples were centrifuged at 3000 × *g* for 10 min. Subsequently, the serum was carefully preserved at −80°C. The sandwich ELISA method was employed to determine the concentrations of serum amylase, lipase, interleukin-6 (IL-6), interleukin-10 (IL-10), tumor necrosis factor-alpha (TNF-*α*), and transforming growth factor-beta (TGF-*β*), using the targeted antibody coated in a 48-well microplate, in strict accordance with the guidelines mentioned in the assay kit.

### 2.6. The TdT-Mediated dUTP Nick-End Labeling (TUNEL) Assay for Detecting Apoptosis in Pancreatic Tissue

First, pancreatic tissue underwent dehydration and deparaffinization, followed by precise repair. The tissue was then washed three times with PBS and incubated with fluorescent TUNEL solution for 1 h. Finally, it was washed three more times with PBS, and the nuclei were stained. Finally, the slides were sealed and stored at a temperature of −20°C, with all the aforementioned steps carried out under optimal dark conditions. The resulting images of rat pancreatic tissue were subjected to scanning using a slice scanner in the green, blue, and mixed wavelength bands and then manually counted to determine the percentage of apoptotic cells with positive expression.

### 2.7. Real-Time Fluorescence Quantitative Polymerase Chain Reaction (RT-qPCR)

First, rat pancreatic tissue was thoroughly homogenized and mixed with 1 mL of Trizol, followed by the addition of protein wash solution and 0.2 mL of chloroform. After genomic DNA removal, a reverse transcription system was prepared to obtain cDNA. The full gene sequence was then retrieved from the NCBI database. Specific primers for each gene were designed using Primer Premier primer design software (Table 2). All primers were expertly designed and synthesized by Shanghai Sangon Biotech and then purified using ULTRAPAGE. Finally, a real-time fluorescence quantitative PCR reaction system was established to determine the relative mRNA expression levels. The relative mRNA expression levels were calculated by 2^−*ΔΔ*CT^.

### 2.8. Western Blot (WB)

The pancreatic tissue was thoroughly homogenized, and proteins were extracted after low-temperature lysis. Protein concentrations were determined using a BCA protein assay kit, allowing for normalization of protein concentrations across groups. After preparing the gel, samples were loaded for electrophoresis, followed by transfer onto a 0.45 μm PVDF membrane. Thereafter, block with the first antibody (dilution ratio: AKT 1:5000; Bax 1:2000; Bcl2 1:2000; Caspase-3 1:2000; PI3K 1:1000; PTEN 1:2000; GAPDH 1:50000), followed by incubation with the second antibody (dilution ratio: 1:5000). Finally, the membrane was placed into an imaging system, where exposure time was adjusted according to signal intensity.

### 2.9. Statistical Analysis

Statistical analyses were performed using SPSS version 25.0 (IBM Corp., Armonk, NY, USA). All data are expressed as mean ± SD and one-way analysis of variance (ANOVA) was used to assess the significance of differences. A *p*-value of <0.05 was considered statistically significant. Additionally, statistical plots in this study were generated using GraphPad Prism 8.0.2 (GraphPad Software, San Diego, USA).

## 3. Results

### 3.1. The miRNA Network Conveyed by hucMSC-Ex

**Figure 1:**
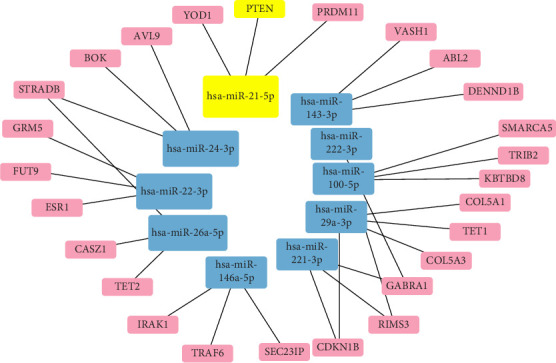
The top 10 miRNAs ordered by relative abundance detected in hucMSC-Ex's absolute quantitative sequencing and their subsequent mRNA targets in a gene interaction network. The size of each box within the schematic depicts the relative content, while the presence of a highlighted yellow core pathway further emphasizes its importance. hucMSC-Ex, human umbilical cord mesenchymal stem cells-derived exosome.

Through the utilization of miRDB, we adeptly assembled an intricate miRNA network conveyed by hucMSC-Ex. Our predictive model enabled the identification of the mRNA targets governed by the top 10 sorted miRNA in GSE 159814, thereby facilitating the creation of a comprehensive miRNA–mRNA gene interaction network (Figure 1). An analysis of our results indicated that hsa-miR-21-5p harbored the highest expression levels and that the activated mRNA PTEN featured prominently among those regulated by hsa-miR-21-5p. Importantly, we tentatively hypothesized that hsa-miR-21-5p is the primary active agent behind the therapeutic effects of hucMSC-Ex, with downstream signaling pathways dependant on the regulation mediated through PTEN.

### 3.2. Identifying hUC-MSCs and hucMSC-Ex

**Figure 2:**
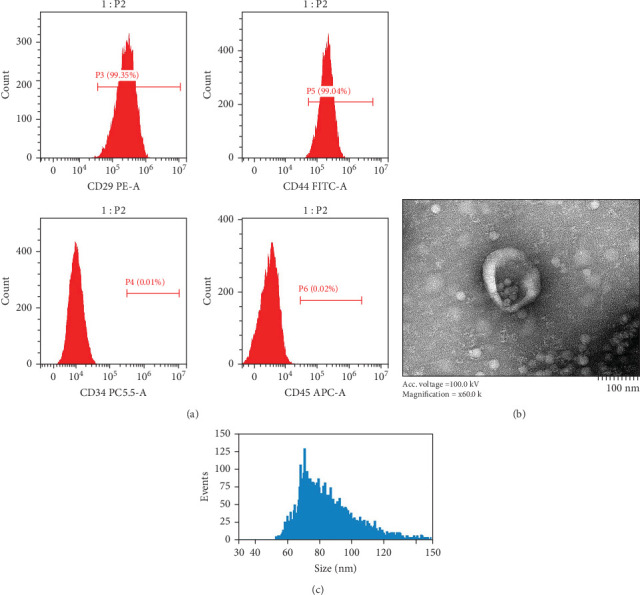
Identifying hUC-MSCs and hucMSC-Ex. (A) the expression levels of stem cells specific surface antigens (CD29 and CD44), and contrasting negative non-stem cells surface antigens (CD34 and CD45). (B) The electron microscopy visualizations of hucMSC-Ex, highlighting their distinct membrane structures. (C) The diverse distribution of diameters and concentrations of hucMSC-Ex was observed through nanoscale particle analysis. hucMSC-Ex, human umbilical cord mesenchymal stem cells-derived exosome; hUC-MSCs, human umbilical cord mesenchymal stem cells.

Our cells of interest were identified by flow cytometry analysis, revealing marked expression of CD29 and CD44 antigens, with concomitant negative expression of CD34 and CD45 markers, indicative of a phenotype synonymous with MSCs (as illustrated in Figure 2A). Upon closer scrutiny through electron microscopy imaging, the distinctive “cup-and-saucer” shape of peripheral membranes enveloping low-density central components, characterizing living cell-secreted membranous vesicles with diameters ranging between 30 and 150 nm, was clearly discernible—thereby underscoring the defining feature of hucMSCs-Ex (as highlighted in Figure 2B). A particle sizing analysis further confirmed the dominant diameter range of extracellular vesicles to lie between 60 and 120 nm (refer to Figure 2C)—thereby reinforcing the identity of hucMSCs-Ex.

### 3.3. Analysis of Pathological Injury of Pancreatic Tissue

**Figure 3:**
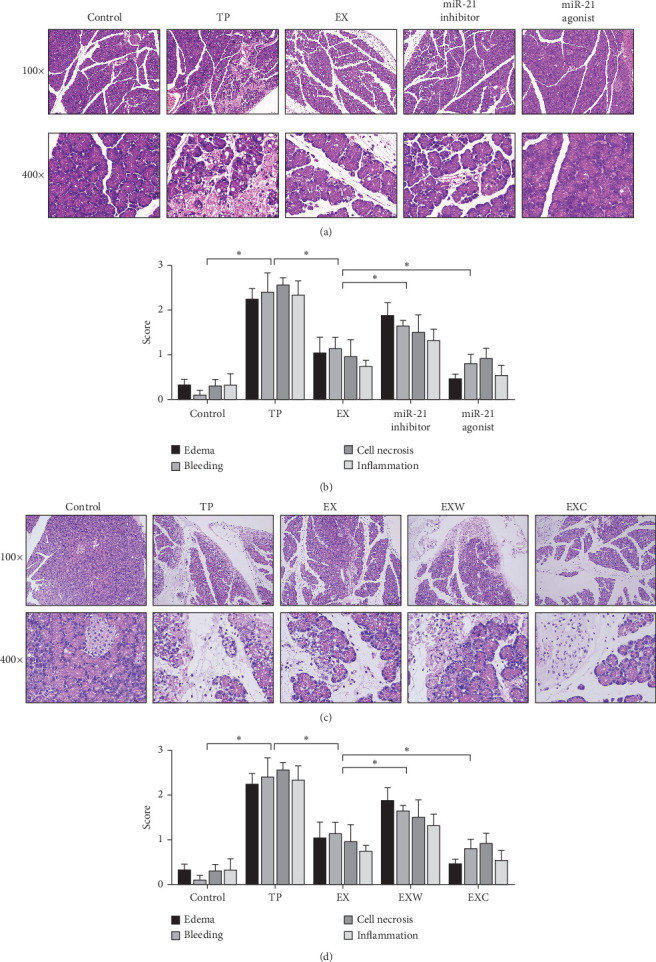
The results of HE and pathology scoring of pancreatic tissue. (A) The first group exhibits the HE staining observed under magnifications of 100 × and 400 ×. (B) Four pathology scores for edema, hemorrhage, necrosis, and inflammatory cell infiltration. (C) The second group exhibits the HE staining observed under magnifications of 100 × and 400 ×. (D) Four pathology scores. *⁣*^*∗*^ Denotes that *p* < 0.05. HE, histological examination.

In this study, one rat in the TP group died, while the rest survived. The HE staining results showed that there was no apparent pathological damage, cell necrosis, or inflammatory cell infiltration in the pancreatic tissues of the control group (Figure 3A,C). However, in the TP group, there was unclear separation of pancreatic lobules, significant widening of intercellular spaces, and visible infiltration of inflammatory cells and edema. In the EX group, some acinar cells were partially atrophied with mild inflammatory cell infiltration. The miR-21 inhibitor group showed nuclear and cytoplasmic disintegration and severe destruction of acinar structures, while the miR-21 agonist group showed relatively clear interlobular ducts and blood vessel structures with only a small amount of inflammatory cell infiltration. The EXW group showed local bleeding spots and visible acinar cell necrosis, while the EXC group showed a relatively intact pancreatic tissue membrane with no apparent bleeding.

In terms of pathological scores, compared to the TP group, the EX group had significantly lower scores in various pathological evaluations (*p* < 0.05) (Figure 3B,D). The EXW and EXC groups both showed significant changes compared to the EX group, with the miR-21 inhibitor and EXW groups showing increased scores, indicating aggravated damage and the miR-21 agonist and EXC groups showing decreased scores, indicating reduced damage.

### 3.4. Biochemical Detection of Serum

**Figure 4:**
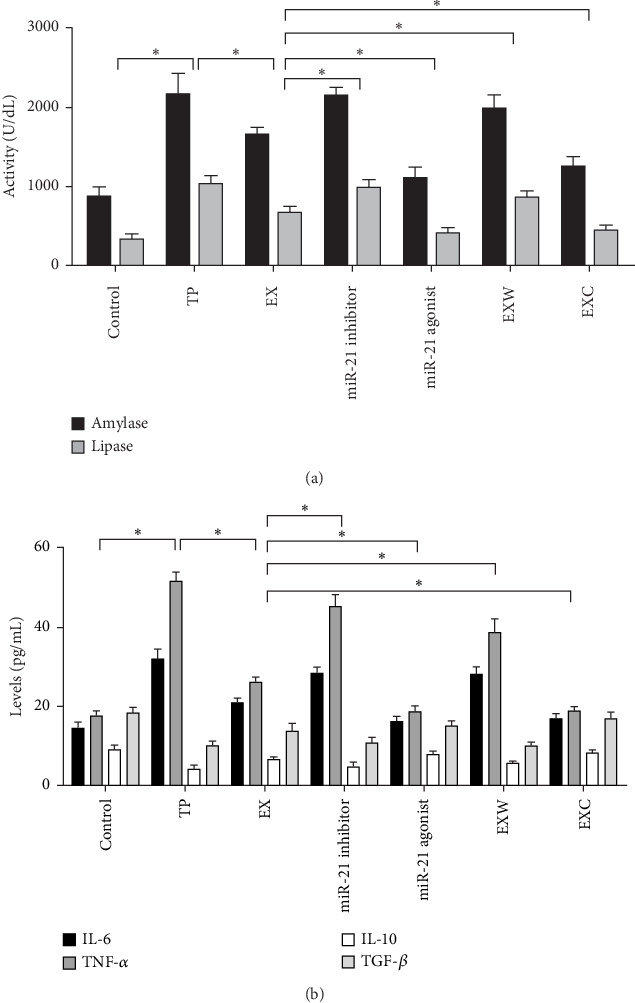
ELISA detection of serum markers. (A) Serum enzymology. (B) Proinflammatory and anti-inflammatory factors. *⁣*^*∗*^ Denotes that *p* < 0.05. ELISA, enzyme-linked immunosorbent assay.

The ELISA test results for serum amylase and lipase expression levels indicate that 24 h after the successful construction of the TP model, the expression level of pancreatic enzymes is significantly higher than that of the control group (*p*  < 0.05), indicating the occurrence of pancreatitis (Figure 4A). Meanwhile, the expression level of pancreatic enzymes in the EX group was significantly lower than that in the TP group (*p*  < 0.05), confirming the therapeutic effect of hucMSC-Ex on TP rats. The therapeutic effect of hucMSC-Ex was inhibited by using a miR-21-5p antagomir, which resulted in a significant increase in pancreatic enzyme expression levels. The treatment effect was significantly enhanced by increasing the expression level of hsa-miR-21-5p using hsa-miR-21-5p agomir, suggesting that hucMSC-Ex exerts its therapeutic effect through the carried hsa-miR-21-5p. In addition, the therapeutic effect in the EXW group was poor, and the pancreatic enzyme level was significantly increased compared to the EX group, but still significantly improved compared to the TP group. The use of PI3K agonist intervention in the EXC group significantly decreased the pancreatic enzyme level compared to the EX group, showing the opposite trend to the EXW group, indicating that hucMSC-Ex regulates the level of serum pancreatic enzymes in rats through PI3K.

In the results of the serum inflammatory factor detection, the trends of proinflammatory factors IL-6 and TNF-*α* in each group were consistent with those of pancreatic enzymes, while anti-inflammatory factors IL-10 and TGF-*β* showed the opposite trend to proinflammatory factors in each group (Figure 4B). This suggests that hucMSC-Ex carrying hsa-miR-21-5p inhibits the inflammatory response by regulating PTEN/PI3K, thereby achieving a therapeutic effect.

### 3.5. The mRNA and Protein Expression Levels of PTEN/PI3K/AKT

**Figure 5:**
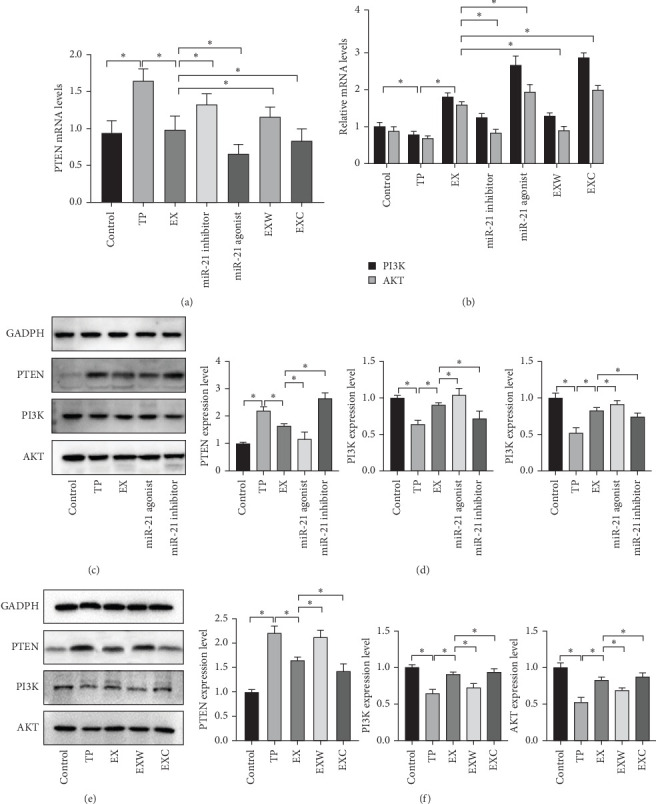
The mRNA and protein expression levels of PTEN/PI3K/AKT. (A) The mRNA expression level of PTEN. (B) The mRNA expression level of PI3K/AKT. (C) The western blot of the first group. (D) Protein expression levels of the first group. (E) The western blot of the second group. (F) Protein expression levels of the second group. *⁣*^*∗*^ Denotes that *p* < 0.05. AKT, serine–threonine kinase; PI3K, phosphoinositide 3-kinase; PTEN, phosphatase and tensin homolog.

The results of PCR detection indicated that mRNA expression levels of PTEN varied significantly between groups, with the TP group displaying the highest levels, suggesting overactivation of PTEN posttrauma. Notably, treatment with hucMSC-Ex led to marked reductions in PTEN expression levels. Further intervention with miR-21 inhibitors and agonists produced observable changes in PTEN expression. Specifically, miR-21-5p inhibitors reduced the inhibitory effect of hucMSC-Ex on PTEN, while miR-21-5p agonists heightened the inhibitory effect of hucMSC-Ex on PTEN (Figure 5A). Administering PI3K agonists and inhibitors on TP rats treated with hucMSC-Ex resulted in the opposite PTEN trend, as PTEN expression levels increased after PI3K inhibition and decreased after PI3K activation. Moreover, the mRNA detection results of PI3K and AKT exhibited an opposite trend to PTEN, thus creating evidence to support that hucMSC-Ex carrying miR-21-5p inhibits PTEN for the excessive activation of PI3K to exert therapeutic effects (Figure 5B).

Further WB testing revealed the protein expression levels among groups in both groups were similar in terms of GADPH expression levels. Observing the protein expression levels of the control group, TP group, and EX group, PTEN expression levels were observed to be substantially higher in the TP group than in the control group, indicating PTEN activation posttrauma. On the other hand, PTEN expression levels were notably lower in the EX group, implying that PTEN expression in pancreatic tissue had been suppressed following hucMSC-Ex treatment (Figure 5C,D). Additionally, PTEN/AKT in the TP and EX groups showcased an opposite trend to that of PTEN, indicating that PTEN changes directly influenced the PI3K/AKT pathway (Figure 5E,F).

In the first group, the miR-21 agonist group showcased a decline in the expression of PTEN, whereas the miR-21 inhibitor group showed an increase in PTEN expression, which suggests that miR-21-5p blocks PTEN translation. The expression of PI3K and AKT, on the other hand, displayed an upsurge in the miR-21 agonist group and a downturn in the miR-21 inhibitor group, indicating significant differences and validating the inhibitory impact of PTEN on PI3K/AKT expression. Moreover, in the second group, PTEN protein expression was observed to be reinforced in EXW groups treated with PI3K inhibitors, while it significantly decreased in EXW groups treated with PI3K activators, reinforcing the notion that PTEN and PI3K have a mutual antagonism.

### 3.6. Apoptosis Detection in Pancreatic Tissue

**Figure 6:**
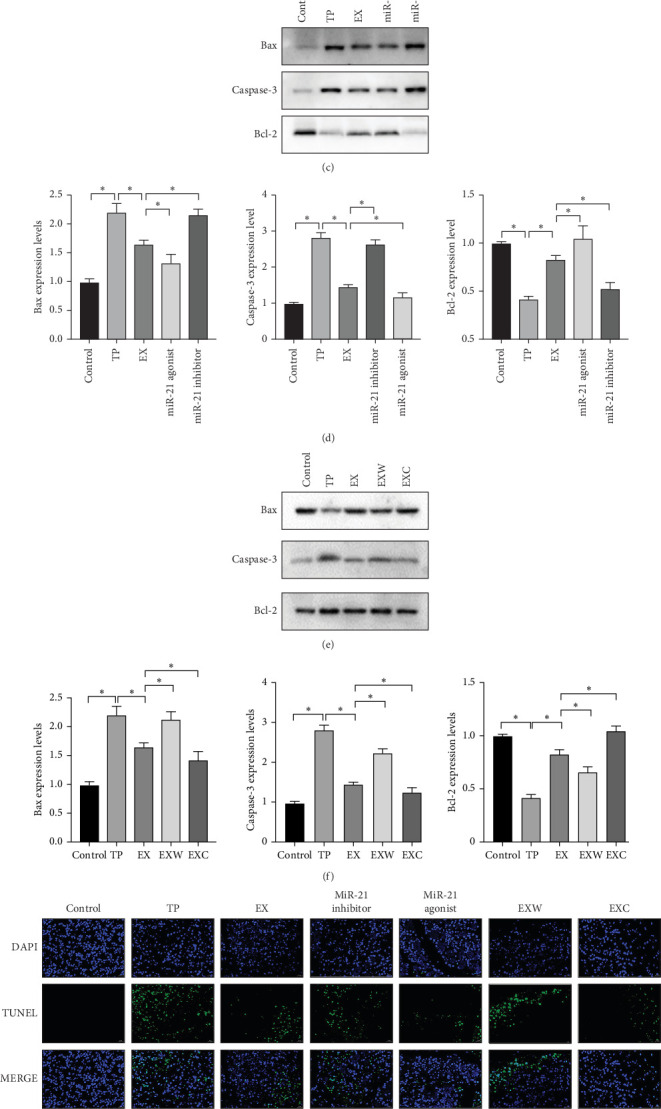
Analysis of apoptosis in pancreatic tissue. (A) The mRNA expression of promote apoptosis. (B) The mRNA expression of Bcl-2. (C) The western blot of the first group. (D) Protein expression levels of the first group. (E) The western blot of the second group. (F) Protein expression levels of the second group. (G) Fluorescence expression of cells. (H) Apoptosis rate.*⁣*^*∗*^ Denotes that *p* < 0.05.

This study investigates apoptosis in pancreatic tissue at the mRNA, protein, and cellular levels. First, mRNA related to apoptosis, such as Bax and Caspase-3, were examined. The results indicate a significant decrease in the expression levels of Bax and Caspase-3, which promote apoptosis in the TP group, whereas in the EX group, the same mRNA expression levels were strengthened (Figure 6A,B). Conversely, the levels of apoptosis-promoting mRNA were increased in the miR-21 inhibitor and EXW group, whereas those in the miR-21 agonist and EXC groups decreased. Furthermore, the expression level of antiapoptotic mRNA Bcl-2 was found to be opposite to that of Bax in all groups, which supports the aforementioned results.

According to the WB results, the proapoptotic proteins Bax and Caspase-3 in the first group were downregulated in the miR-21 agonist group, while their expression was upregulated in the miR-21 inhibitor group (Figure 6C,D). Conversely, the antiapoptotic protein Bcl-2 revealed an opposite trend in these two groups, suggesting that an excessive amount of hsa-miR-21-5p directly inhibits apoptosis. In the second group, the expression level of antiapoptotic protein Bcl-2 in the EXW group was reduced, whereas the expression level of Bcl-2 in the EXC group was increased (Figure 6E,F). The trend of proapoptotic proteins was opposite to that of Bcl-2, indicating that the activation of PI3K directly inhibits apoptosis.

Finally, the TUNEL assay was employed in this study to observe cellular apoptosis under mixed light (MERGE), blue light (diamidinophenylindole [DAPI]), and green light (TUNEL) conditions (Figure 6G). The results indicate a significant increase in the fluorescence intensity of acinar cells in the TP group compared to the control group, suggesting a widespread acinar cell apoptosis following pancreatic trauma. However, the acinar cell apoptosis rate significantly decreased after hucMSC-Ex treatment, with a significant statistical difference (*p* < 0.05) (Figure 6H). Moreover, compared to the EX group, the miR-21 inhibitor and miR-21 agonist groups exhibited increased and decreased cellular apoptosis rates, respectively, indicating that hucMSC-Ex suppresses acinar cell apoptosis by carrying hsa-miR-21-5p. Additionally, the apoptosis rate in the EXW group was significantly higher than that in the EX group, suggesting that PI3K inhibition enhances acinar cell apoptosis. Conversely, the apoptosis rate in the EXC group was significantly lower than that in the EX group, indicating that PI3K activation inhibits acinar cell apoptosis.

## 4. Discussion

As a distinct form of acute pancreatitis (AP), TP is characterized by heavy damage, difficult-to-control inflammatory reactions, and a “cascade amplification effect” [23]. The essence of this process is the uncontrolled self-digestion of the pancreas caused by uncontrolled pancreatic enzymes, resulting in feedback damage that is difficult to control. Therefore, timely blocking of excessive death of acinar cells helps prevent pancreatic enzyme leakage, reduce edema and inflammatory reactions [24]. Currently, treatment for TP is mainly based on fluid supplementation, life support, and timely surgical removal of damaged tissue, lacking targeted therapy based on precision medicine concepts [25, 26]. In recent years, research into the therapeutic potential of exosomes derived from MSCs for various traumatic diseases has intensified, highlighting their potential in clinical transformative therapy. Our previous study demonstrated that both hUC-MSCs and hucMSC-Ex could localize to damaged pancreatic sites. Subsequent analysis of pancreatic tissue damage indicated comparable therapeutic efficacy between the two, further reinforcing the consistent benefits of stem cell-derived exosomes in treating traumatic diseases [6, 7].

The miRNA in the exosomes derived from stem cells is the most abundant bioactive substance [27–29]. Previous studies have demonstrated that the miRNA in the exosomes from MSCs plays a critical role in cardiovascular protection, regeneration, and repair, as well as in inhibiting apoptosis and fibrosis of cardiac cells [30, 31]. To further clarify the molecular effects of hucMSC-Ex in TP therapy, we have combined the quantitative miRNA sequencing results of hucMSC-Ex in the GEO database and determined that hsa-miR-21-5p has the highest abundance. Moreover, we have constructed a miRNA-mRNA interaction network and found that PTEN is a core factor that is mainly suppressed by hsa-miR-21-5p carried by hucMSC-Ex to exert a therapeutic effect. PCR analysis further confirmed that PTEN expression in the pancreatic tissues of TP rats was significantly reduced following hUCMSC-Ex treatment, suggesting that hsa-miR-21-5p modulates PTEN expression. According to related research, PTEN primarily acts as an antagonist of PI3K, influencing AKT phosphorylation and thereby impacting cell proliferation, apoptosis, and metabolism. Similarly, in our study, both mRNA and protein detection showed that PTEN and PI3K had opposite trends in each group, supporting the views of related research. Therefore, the focus of our study is on the hsa-miR-21-5p/PTEN/PI3K signaling pathway.

In this study, there were two research groups aimed at exploring the therapeutic effects of hsa-miR-21-5p and PTEN/PI3K on TP. In the first group, hsa-miR-21-5p's angtagomir and agomir reagents were used to specifically inhibit or activate hsa-miR-21-5p. It was found that inhibiting hsa-miR-21-5p decreases hucMSC-Ex's therapeutic effect on TP, including worsening of the pathological injury in pancreatic tissue, increase in pancreatic enzyme levels, and enhanced inflammatory reactions. These results suggested that hsa-miR-21-5p had a therapeutic effect on acinar cells in the rat pancreas following injury. Specifically, the pancreatic tissue in the TP group exhibited typical pathological damage, including indistinct lobular boundaries, significantly expanded intercellular spaces, extensive inflammatory cell infiltration, and severe edema. In contrast, the EX group showed markedly reduced acinar cell injury, with significantly lower scores for pancreatic edema, hemorrhage, cell necrosis, and inflammatory cell infiltration, indicating a strong therapeutic effect of hucMSC-Ex on TP. However, this therapeutic effect was significantly blocked by the miR-21 inhibitor, as evidenced by much higher pancreatic pathology scores in the miR-21 inhibitor group compared to the EX group. Additionally, WB results showed a marked increase in PTEN expression in the miR-21 inhibitor group, suggesting that the effect of exosome-delivered miR-21 in inhibiting PTEN translation was blocked. Conversely, the miR-21 agonist group effectively elevated miR-21 levels in the pancreatic injury region, further reducing PTEN translation compared to the EX group. This rescue experiment highlights the regulatory role of hucMSC-Ex-derived miR-21 in controlling PTEN expression.

Further, TUNEL results showed that inhibiting hsa-miR-21-5p led to increased acinar cell apoptosis, which weakened the therapeutic effect of hucMSC-Ex. Another study found that the exosomes derived from bone marrow MSCs, when cocultured with cardiomyocytes, can antagonize PTEN through carrying specific microRNAs to activate the PI3K/AKT pathway and thereby inhibit apoptotic injury of myocardial cells [32]. Thus, hsa-miR-21-5p carried by hucMSC-Ex was identified as the key biological active substance of the exosomes derived from hUC-MSCs for the therapeutic effect of TP.

The second group of this study further targets the downstream target mRNA of hsa-miR-21-5p, namely PTEN/PI3K. By inhibiting or activating PI3K using wortmannin and 1,3-dicaffeoylquinic acid, it was observed that inhibiting PI3K (i.e., activating PTEN) would exacerbate the tissue damage caused by hucMSC-Ex treatment in TP rats while activating PI3K would enhance the therapeutic effect of hucMSC-Ex. Moreover, activating PI3K would directly inhibit cell apoptosis. In an in vitro experiment, it was found that activating PI3K/AKT would alleviate oxidative stress, inflammation, and apoptosis induced by hypoxia, thereby mitigating the damage to proximal renal tubular epithelial cells caused by hypoxia [33]. An animal experiment constructing a cerebral infarction rat model found that PI3K activation would exert a therapeutic effect on cerebral infarction rats by reducing neuron apoptosis [34]. After being activated, AKT would be completely activated by phosphorylation of PI3K, resulting in increased AKT activity, changes in the cell cycle, and inhibition of cell apoptosis [35, 36]. In the process of inhibiting acinus cell apoptosis through PTEN/PI3K/AKT, the core link lies in the activation of AKT, which exerts an antiapoptotic effect by phosphorylating target proteins through multiple downstream pathways [37–41]. First, AKT promotes the release of NF-*κ*B from the cytoplasm, thereby facilitating its nuclear translocation and promoting cell survival. In addition, at the onset of apoptosis, AKT phosphorylates Bcl-2, which impedes the process. In our research, we observed that activation of AKT by a PI3K agonist led to increased expression levels of Bcl-2. Additionally, AKT was found to inhibit the activation of caspase-9, which initiates apoptotic cascades. Moreover, through phosphorylation of the tumor suppressor protein p53, AKT induced cell cycle arrest, facilitating DNA repair and preventing apoptosis. Therefore, our findings suggest that hsa-miR-21-5p in the second group exerts a therapeutic effect via the PTEN/PI3K pathway.

**Figure 7:**
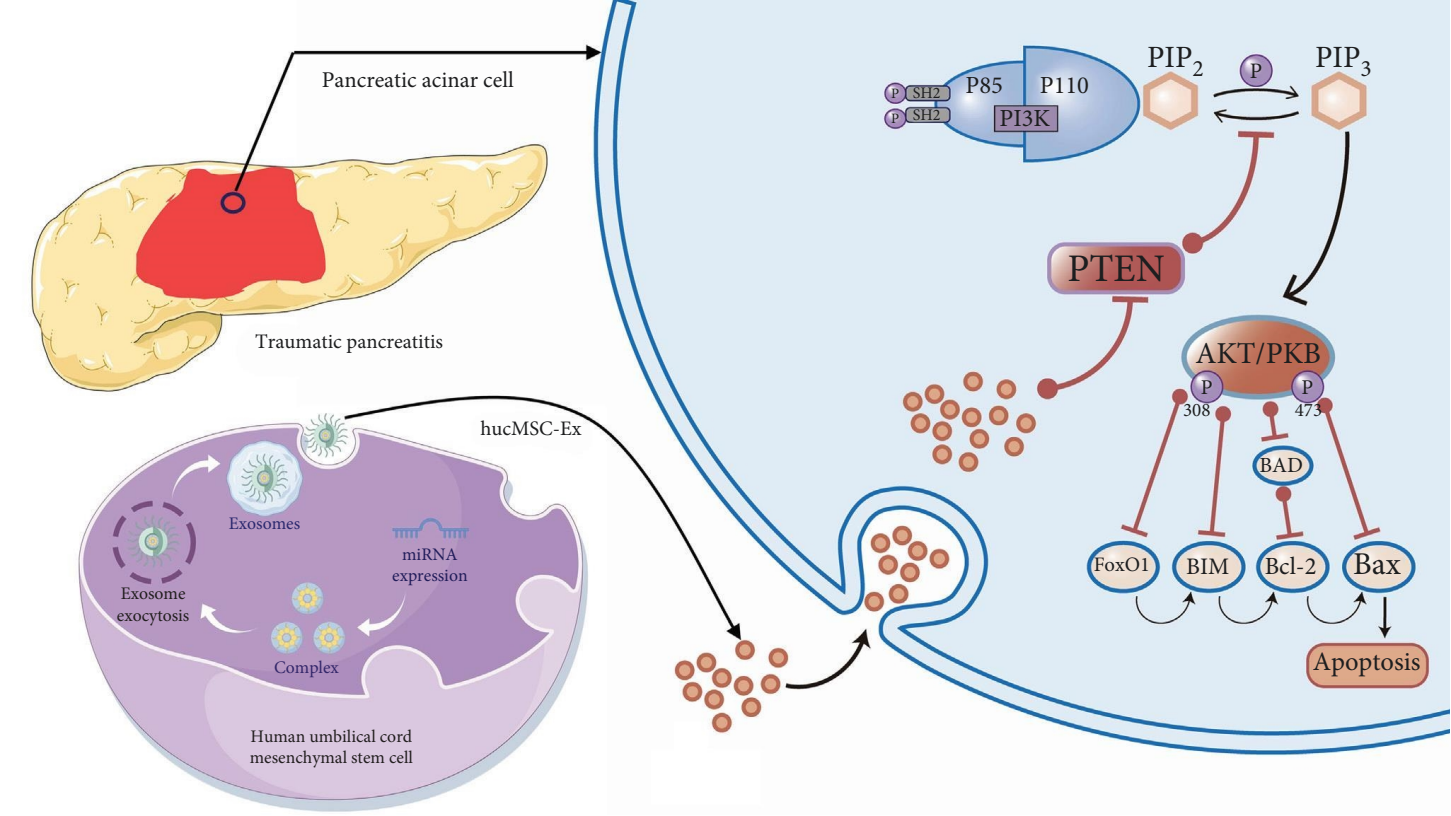
The treatment mechanism of hucMSC-Ex against TP. Delivery by exosomes, hsa-miR-21-5p, penetrates the plasma membranes and orchestrates a potent antiapoptotic response in pancreatic acinar cells of TP rats via intricate regulation of PTEN/PI3K signaling pathways. hucMSC-Ex, human umbilical cord mesenchymal stem cells-derived exosome; PI3K, phosphoinositide 3-kinase; PTEN, phosphatase and tensin homolog; TP, traumatic pancreatitis.

In this study, we screened the core miRNAs and their downstream target genes based on the miRNA sequencing results of hucMSC-Ex. By regulating hsa-miR-21-5p and PTEN/PI3K, respectively, we investigated the therapeutic effects and potential molecular mechanisms of hucMSC-Ex on TP (Figure 7).

This study has several limitations. First, we selected adult male rats to maintain stable hormone levels, but future research should investigate potential sex differences by including female rats. Second, due to the challenges of replicating trauma models in vitro, this study did not explore the mechanisms at the cellular level. Finally, as hucMSC-Ex are complex biological vesicles, interactions between miRNAs were not considered. Future studies analyzing the thousands of miRNAs contained in hucMSC-Ex will help to further elucidate their therapeutic potential from a mechanistic perspective. Additionally, multiomics analysis based on stem cell-derived exosomes, combined with targeted regulation of noncoding RNAs and protein carriers, will enhance their therapeutic efficacy and establish a theoretical foundation for clinical applications.

## 5. Conclusion

In summary, this study found that hucMSC-Ex directly suppresses the translation of PTEN through the carried hsa-miR-21-5p, thereby affecting the PI3K/AKT signaling pathway. This results in decreased inflammation and the inhibition of acinar cell apoptosis by regulating pancreas enzyme leakage, leading to a therapeutic effect on TP.

## Figures and Tables

**Table 1:** Detailed modeling information for each group.

Group naming	Intervention before modeling	Molding	Treatment after modeling
Control group	—	—	—

TP group	—	Trauma	Intravenous injection of 1 mL saline.

EX group	—	Trauma	Within 0.5 h after modeling, intravenous injection of 1 mL hucMSC-EX (20 μg/mL; 10 μg/100 g).

miR-21 inhibitor group	Intravenous injection of 200 μL hsa-miR-21-5p angtagomir, 2 h prior to modeling.	Trauma	The same hucMSC-EX therapy

miR-21 agonist group	Intravenous injection of 200 μL hsa-miR-21-5p agomir, 2 h prior to modeling.	Trauma	The same hucMSC-EX therapy

EXW	Intravenous injection of 500 μL wortmannin, 2 h prior to modeling.	Trauma	The same hucMSC-EX therapy

EXC	Intravenous injection of 500 μL 1,3-dicaffeoylquinic acid, 2 h prior to modeling.	Trauma	The same hucMSC-EX therapy

**Table 2:** RT-qPCR primer sequences.

Genes	Primer sequences
GADPH	F: ACAGCAACAGGGTGGTGGAC
	R: TTTGAGGGTGCAGCGAACTT
Bax	F: AGACACCTGAGCTGACCTTGGAG
	R: TTCATCGCCAATTCGCCTGAGAC
Bcl-2	F: TAGAGAGCGTCAACAGGGAGATG
	R: GTGCAGATGCCGGTTCAGGTAC
Caspase-3	F: GCGGTATTGAGACAGACAGTGGAAC
	R: AACCATGACCCGTCCCTTGAATTTC
PTEN	F: TTTGAAGACCATAACCCACCACAGC
	R: CATTACACCAGTCCGTCCTTTCCC
PI3K	F: GCTGTTGATAGACCACCGCTTCC
	R: TGCCCTGTTCCTCTGCCTTCC
AKT	F: CACAGGTCGCTACTATGCCATGAAG
	R: GCAGGACACGGTTCTCAGTAAGC

## Data Availability

The data that support the findings of this study are available from the corresponding author upon reasonable request.

## Nomenclature

TP: Traumatic pancreatitis
AP: Acute pancreatitis
hucMSC-Exs: Human umbilical cord mesenchymal stem cells-exosomes
GEO: Gene Expression Omnibus
PTEN: Phosphatase and tensin homolog
PI3K: Phosphoinositide 3-kinase
AKT: Serine–threonine kinase
HE: Histological examination
SD: Sprague-Dawley
ELISA: Enzyme-linked immunosorbent assay
TUNEL: TdT-mediated dUTP Nick-End Labeling
DAPI: Diamidinophenylindole
ANOVA: One-way analysis of variance.

## References

[1] Shibahashi K., Sugiyama K., Kuwahara Y., Ishida T., Okura Y., Hamabe Y. (2020). Epidemiological State, Predictive Model for Mortality, and Optimal Management Strategy for Pancreatic Injury: A Multicentre Nationwide Cohort Study. *Injury*.

[2] Pavlidis E. T., Psarras K., Symeonidis N. G., Geropoulos G., Pavlidis T. E. (2022). Indications for the Surgical Management of Pancreatic Trauma: An Update. *World Journal of Gastrointestinal Surgery*.

[3] Zhong Z., Tian Y., Luo X., Zou J., Wu L., Tian J. (2021). Extracellular Vesicles Derived From Human Umbilical Cord Mesenchymal Stem Cells Protect Against DOX-Induced Heart Failure Through the miR-100-5p/NOX4 Pathway. *Frontiers in Bioengineering and Biotechnology*.

[4] Wei Q., Wang Y., Ma K. (2022). Extracellular Vesicles From Human Umbilical Cord Mesenchymal Stem Cells Facilitate Diabetic Wound Healing Through MiR-17-5p-Mediated Enhancement of Angiogenesis. *Stem Cell Reviews and Reports*.

[5] Chen X., Su C., Wei Q., Sun H., Xie J., Nong G. (2022). Exosomes Derived From Human Umbilical Cord Mesenchymal Stem Cells Alleviate Diffuse Alveolar Hemorrhage Associated With Systemic Lupus Erythematosus in Mice by Promoting M2 Macrophage Polarization via the microRNA-146a-5p/NOTCH1 Axis. *Immunological Investigations*.

[6] Han L., Zhao Z., Chen X. (2022). Human Umbilical Cord Mesenchymal Stem Cells-Derived Exosomes for Treating Traumatic Pancreatitis in Rats. *Stem Cell Research & Therapy*.

[7] Li H., Zhirong Z., Shibo Z. (2023). The Effects of Umbilical Cord Mesenchymal Stem Cells on Traumatic Pancreatitis in Rats. *Digestive Diseases and Sciences*.

[8] Horie S., Gonzalez H., Brady J. (2021). Fresh and Cryopreserved Human Umbilical-Cord-Derived Mesenchymal Stromal Cells Attenuate Injury and Enhance Resolution and Repair Following Ventilation-Induced Lung Injury. *International Journal of Molecular Sciences*.

[9] Cui L., Luo W., Jiang W. (2022). Human Umbilical Cord Mesenchymal Stem Cell-Derived Exosomes Promote Neurological Function Recovery in Rat After Traumatic Brain Injury by Inhibiting the Activation of Microglia and Astrocyte. *Regenerative Therapy*.

[10] Zhang L., Song Y., Chen L. (2020). MiR-20a-Containing Exosomes from Umbilical Cord Mesenchymal Stem Cells Alleviates Liver Ischemia/Reperfusion Injury. *Journal of Cellular Physiology*.

[11] Beylerli O., Tamrazov R., Gareev I. (2023). Role of Exosomal ncRNAs in Traumatic Brain Injury. *Non-Coding RNA Research*.

[12] Ghafouri-Fard S., Shoorei H., Dong P. (2023). Emerging Functions and Clinical Applications of Exosomal MicroRNAs in Diseases. *Non-Coding RNA Research*.

[13] Li Y., Tang X., Wang B., Chen M., Zheng J., Chang K. (2024). Current Landscape of Exosomal Non-Coding RNAs in Prostate Cancer: Modulators and Biomarkers. *Non-Coding RNA Research*.

[14] Wang Y., Lai X., Wu D., Liu B., Wang N., Rong L. (2021). Umbilical Mesenchymal Stem Cell-Derived Exosomes Facilitate Spinal Cord Functional Recovery Through the miR-199a-3p/145-5p-Mediated NGF/TrkA Signaling Pathway in Rats. *Stem Cell Research & Therapy*.

[15] Zhang F., Gou Z., Zhou Y. (2022). MicroRNA-21-5p Agomir Inhibits Apoptosis of Oligodendrocyte Precursor Cell and Attenuates White Matter Injury in Neonatal Rats. *Brain Research Bulletin*.

[16] Lv X., Liang J., Wang Z. (2024). MiR-21-5p Reduces Apoptosis and Inflammation in Rats With Spinal Cord Injury Through PI3K/AKT Pathway. *Panminerva Medica*.

[17] Liu Y. P., Tian M. Y., Yang Y. D. (2022). Schwann Cells-Derived Exosomal miR-21 Participates in High Glucose Regulation of Neurite Outgrowth. *iScience*.

[18] Ranganna K., Selvam C., Shivachar A., Yousefipour Z. (2020). Histone Deacetylase Inhibitors as Multitarget-Directed Epi-Drugs in Blocking PI3K Oncogenic Signaling: A Polypharmacology Approach. *International Journal of Molecular Sciences*.

[19] Chelh I., Meunier B., Picard B. (2009). Molecular Profiles of Quadriceps Muscle in Myostatin-Null Mice Reveal PI3K and Apoptotic Pathways as Myostatin Targets. *BMC Genomics*.

[20] Zhao Q., Huang L., Qin G. (2021). Cancer-Associated Fibroblasts Induce Monocytic Myeloid-Derived Suppressor Cell Generation via IL-6/Exosomal miR-21-Activated STAT3 Signaling to Promote Cisplatin Resistance in Esophageal Squamous Cell Carcinoma. *Cancer Letters*.

[21] Dai R., Chen G., Huang Z. (2012). Establishment and Characteristics of an Animal Model for Isolated Pancreatic Trauma. *Journal of Trauma and Acute Care Surgery*.

[22] Schmidt J., Rattner D. W., Lewandrowski K. (1992). A Better Model of Acute Pancreatitis for Evaluating Therapy. *Annals of Surgery*.

[23] Wang B., Lin W. (2020). Edaravone Protects Against Pancreatic and Intestinal Injury after Acute Pancreatitis via Nuclear Factor-*κ*B Signaling in Mice. *Biological and Pharmaceutical Bulletin*.

[24] Kanno H., Hirakawa Y., Yasunaga M. (2021). Successful Nonoperative Management by Endoscopic and Percutaneous Drainage for Penetrating Pancreatic Duct Injury: A Case Report. *Journal of Medical Case Reports*.

[25] Degiannis E., Glapa M., Loukogeorgakis S. P., Smith M. D. (2008). Management of Pancreatic Trauma. *Injury*.

[26] Petrone P., Moral Álvarez S., González Pérez M., Ceballos Esparragón J., Marini C. P. (2017). Pancreatic Trauma: Management and Literature Review. *Cirugia Espanola*.

[27] Douvris A., Viñas J., Burns K. D. (2022). miRNA-486-5p: Signaling Targets and Role in Non-Malignant Disease. *Cellular and Molecular Life Sciences*.

[28] Ding Y., Cui Y., Hou Y., Nie H. (2020). Bone Marrow Mesenchymal Stem Cell-Conditioned Medium Facilitates Fluid Resolution via miR-214-Activating Epithelial Sodium Channels. *MedComm*.

[29] Zhang D., Wu Y., Li Z. (2021). MiR-144-5p, an Exosomal miRNA From Bone Marrow-Derived Macrophage in Type 2 Diabetes, Impairs Bone Fracture Healing via Targeting Smad1. *Journal of Nanobiotechnology*.

[30] Drommelschmidt K., Serdar M., Bendix I. (2017). Mesenchymal Stem Cell-Derived Extracellular Vesicles Ameliorate Inflammation-Induced Preterm Brain Injury. *Brain, Behavior, and Immunity*.

[31] Karbasiafshar C., Sellke F. W., Abid M. R. (2021). Mesenchymal Stem Cell-Derived Extracellular Vesicles in the Failing Heart: Past, Present, and Future. *American Journal of Physiology-Heart and Circulatory Physiology*.

[32] Sun X.-H., Wang X., Zhang Y., Hui J. (2019). Exosomes of Bone-Marrow Stromal Cells Inhibit Cardiomyocyte Apoptosis Under Ischemic and Hypoxic Conditions via miR-486-5p Targeting the PTEN/PI3K/AKT Signaling Pathway. *Thrombosis Research*.

[33] Zhang B. H., Liu H., Yuan Y. (2021). Knockdown of TRIM8 Protects HK-2 Cells Against Hypoxia/Reoxygenation-Induced Injury by Inhibiting Oxidative Stress-Mediated Apoptosis and Pyroptosis via PI3K/Akt Signal Pathway. *Drug Design, Development and Therapy*.

[34] Chen J., Zhang W., Wu Y. Q., Chen H., Zhao J. F. (2019). LncRNA SNHG1 Inhibits Neuronal Apoptosis in Cerebral Infarction Rats Through PI3K/Akt Signaling Pathway. *European Review for Medical and Pharmacological Sciences*.

[35] Shan J., Al-Muftah M. A., Al-Kowari M. K. (2019). Targeting Wnt/EZH2/microRNA-708 Signaling Pathway Inhibits Neuroendocrine Differentiation in Prostate Cancer. *Cell Death Discovery*.

[36] Facchini G., Rossetti S., Cavaliere C. (2018). Exploring the Molecular Aspects Associated With Testicular Germ Cell Tumors: A Review. *Oncotarget*.

[37] Zhao Y., Liu X., Ding C., Gu Y., Liu W. (2021). Dihydromyricetin Reverses Thioacetamide-Induced Liver Fibrosis Through Inhibiting NF-*κ*B-Mediated Inflammation and TGF-*β*1-Regulated of PI3K/Akt Signaling Pathway. *Frontiers in Pharmacology*.

[38] Chen K., Jiao J., Xue J. (2020). Ginsenoside CK Induces Apoptosis and Suppresses Proliferation and Invasion of Human Osteosarcoma Cells Through the PI3K/mTOR/p70S6K1 Pathway. *Oncology Reports*.

[39] Dailey W., Shunemann R., Yang F. (2021). Differences in Activation of Intracellular Signaling in Primary Human Retinal Endothelial Cells Between Isoforms of VEGFA 165. *Molecular Vision*.

[40] Xie L. Y., Yang Z., Wang Y. (2022). 1-*O*-Actylbritannilactone Ameliorates Alcohol-Induced Hepatotoxicity Through Regulation of ROS/Akt/NF-*κ*B-Mediated Apoptosis and Inflammation. *ACS Omega*.

[41] Habibian J., Ferguson B. S. (2019). The Crosstalk Between Acetylation and Phosphorylation: Emerging New Roles for HDAC Inhibitors in the Heart. *International Journal of Molecular Sciences*.

